# Energy metabolism and behavior in the corticotropin-releasing factor family of peptides

**DOI:** 10.3389/fnins.2015.00122

**Published:** 2015-04-13

**Authors:** James A. Carr, David A. Lovejoy

**Affiliations:** ^1^Department of Biological Sciences, Texas Tech UniversityLubbock, TX, USA; ^2^Department of Cell and Systems Biology, University of TorontoToronto, ON, Canada

**Keywords:** hypothalamus, stress, evolution, anorexia, anxiety, fishes, mammals, CRF

This year, 2015, will mark the sixtieth anniversary of the seminal work by Guillemin and Rosenberg (1955) and Schally and Saffran (1955) which, along with the earlier work from Geoffrey Harris' lab, initiated the search for an adrenocorticotropin releasing factor that culminated with the discovery of corticotropin-releasing factor (CRF) in 1981 by Wylie Vale's laboratory (Vale et al., 1981). Since the 1980s, the CRF story has had many twists and turns from the finding that CRF and its receptors are located in many extra-hypothalamic brain areas and extending to the discovery that two genome duplications expanded the CRF peptide family hundreds of millions of years ago. Because of the early metazoan ancestry of CRF, this peptide has become ensconced in a number physiological processes. We now know that stress in vertebrates is comprised of a complex set of physiological actions that regulate organismal metabolism to promote protection of the individual and progeny from life-threatening situations. This stress response likely evolved before the earliest multicellular organisms yet has been retained throughout metazoan evolution. Vertebrates, in particular, have developed some of the most complex stress-regulating mechanisms. Integral to this stress response is CRF, and its paralogue, urocortin (urotensin-I/sauvagine) and a parallel paralogous lineage consisting of urocortin 2 and 3.

Six review papers in this volume summarize some of the latest research on the role of CRF and urocortin (UCN) peptides in modulating behavior during stress. In addition, two new research studies present data supporting an ancient role for CRF/UCN receptors in social behavior and feeding, while two additional papers explore the role of extrahypothalamic CRF neurons in behavioral and endocrine responses.

A consistent theme running through this set of papers is that many of the effects of CRF/UCN peptides on physiology and behavior are evolutionarily conserved. In their contribution, Chen et al. (2013) examine the evolutionary origins of the tenuerin C-terminal associated peptides (TCAP), peptides which may have predated the origin of the CRF peptide family. Chen et al. (2013) review data on the neuromodulatory actions of TCAP-1 and both its direct and indirect effects as an indirect modulator of CRF intracellular signaling.

Feeding and intake of nutrients is essential for the success of any species. A stress-response, as regulated by the CRF family of peptides, generally has suppressive actions on appetite and digestion in order to shunt energy from a parasympathetic need to arousal and sympathetic requirements. From an evolutionary point of view this may be an adaptive response when an animal is threatened by a threat in its environment, however there are obvious energetic costs if anorexia is prolonged. As the papers by Matsuda (2013) and Ortega et al. (2013) indicate, CRF inhibition of appetite is a phylogenetically ancient response based on data in fishes. Matsuda (2013) summarizes the data on CRF and appetite in fish and points out the complexities of dissociating specific effects on appetite from the broader impact of CRF peptides on anxiety and locomotor behavior. Ortega et al. (2013) present new data in rainbow trout (*Oncorhyncus mykiss*) suggesting that the anorexic effects of serotonin may be mediated by CRF- and/or urotensin I receptors. Stengel and Taché (2014) provide an extensive and critical look at CRF/UCN peptide signaling via CRF R1/2 receptors in feeding behavior in mammals. These authors not only summarize the latest studies on the central actions of CRF peptides related to food intake in the context of stress adaptation but also look at the interesting possibility that peripheral receptors also may be involved. Stengel and Taché (2014) also summarize studies on CRF receptor single nucleotide polymorphisms (snps) that are associated with obesity.

Emerging evidence from comparative and preclinical studies indicates that CRF may play an adaptive role in modulating social behavior. In vertebrates, social stress is one of the greatest forms of stressors. Prolonged and intense social stress can affect nutrient acquisition and reproduction thus compromising both the health of the individual and the population. In their paper, Solomon-Lane et al. (2013) review the literature linking the hypothalamus-pituitary-interrenal axis to socially-mediated sex change in fishes and provide new data on the role of socially mediated sex change in the blue banded goby (*Lythyrpnus dalli*). Backström and Winberg (2013) take a broad comparative approach to examine the role of the HPI/HPA axis on social stress while Hostetler and Ryabinin (2013) provide an extensive review of the role (s) that central CRF pathways play in social and particularly, reproductive and courtship behavior. Interestingly, a number of authors have commented on the close relationship between the CRF system and the regulation of 5-HT, a neurotransmitter whose evolutionary origins predate multicellular organisms.

Elaboration of the central nervous system is a hallmark of vertebrate morphology and success. It is not surprising, therefore, that the CRF peptide family is associated with numerous neurological circuits regulating most behaviors. The role of CRF and its regulation of pituitary ACTH have been well covered in the past. However, several of the papers in this volume touch on the involvement of CRF/UCN peptides targeting receptors in extra hypothalamic brain areas. These studies collectively highlight the diverse phenotypes of cells producing CRF within the CNS. Fox and Lowry (2013) review the interaction between CRF- and 5-HT-producing neurons in the dorsal raphe nucleus, the major site for 5-HT production within the brain. Dabrowska et al. (2013) provide evidence that CRF neurons in the BNST and PVN are phenotypically different based upon their physiological response to local neurotransmitter release and whether they also synthesize GABA or glutamate and may have either inhibitory or excitatory actions, respectively. Callahan et al. (2013) use RNAi techniques to locally knockdown CRF expression in the central nucleus of the amygdala. Interestingly, disruption of CRF synthesis in this brain area interferes with stress-induced HPA activity and the expression of anxiety-like behaviors during stress.

In summary, papers within this compendium illustrate the long evolutionary history of the CRF family of peptides and their importance to organismal physiology and behavior. Despite the achievements over the last 60 years, there remains much to learn about the role of the CRF family of peptides in the physiology and pathology of vertebrates.

## Conflict of interest statement

The authors declare that the research was conducted in the absence of any commercial or financial relationships that could be construed as a potential conflict of interest.
